# Mitochondrial **β**-oxidation of adipose-derived fatty acids by osteoblasts fuels parathyroid hormone–induced bone formation

**DOI:** 10.1172/jci.insight.165604

**Published:** 2023-02-02

**Authors:** Nathalie S. Alekos, Priyanka Kushwaha, Soohyun P. Kim, Zhu Li, Abdullah Abood, Naomi Dirckx, Susan Aja, Joe Kodama, Jean G. Garcia-Diaz, Satoru Otsuru, Elizabeth Rendina-Ruedy, Michael J. Wolfgang, Ryan C. Riddle

**Affiliations:** 1 Department of Orthopaedics, University of Maryland School of Medicine, Baltimore, Maryland, USA.; 2 Graduate Program in Cellular and Molecular Medicine, Johns Hopkins University School of Medicine, Baltimore, Maryland, USA.; 3 Division of Rheumatology, Inflammation, and Immunity, Department of Medicine, Brigham and Women’s Hospital, Boston, Massachusetts, USA.; 4 Department of Pathology, Johns Hopkins University School of Medicine, Baltimore, Maryland, USA.; 5 Center for Public Health Genomics, School of Medicine, University of Virginia, Charlottesville, Virginia, USA.; 6 Center for Metabolism and Obesity Research, Johns Hopkins University School of Medicine, Baltimore, Maryland, USA.; 7 Department of Medicine and Division of Clinical Pharmacology, Vanderbilt University Medical Center, Nashville, Tennessee, USA.; 8 Department of Biological Chemistry, Johns Hopkins University School of Medicine, Baltimore, Maryland, USA.; 9 Research & Development Service, Baltimore Veterans Administration Medical Center, Baltimore, Maryland, USA.

**Keywords:** Bone biology, Adipose tissue, Fatty acid oxidation, Osteoporosis

## Abstract

The energetic costs of bone formation require osteoblasts to coordinate their activities with tissues, like adipose, that can supply energy-dense macronutrients. In the case of intermittent parathyroid hormone (PTH) treatment, a strategy used to reduce fracture risk, bone formation is preceded by a change in systemic lipid homeostasis. To investigate the requirement for fatty acid oxidation by osteoblasts during PTH-induced bone formation, we subjected mice with osteoblast-specific deficiency of mitochondrial long-chain β-oxidation as well as mice with adipocyte-specific deficiency for the PTH receptor or adipose triglyceride lipase to an anabolic treatment regimen. PTH increased the release of fatty acids from adipocytes and β-oxidation by osteoblasts, while the genetic mouse models were resistant to the hormone’s anabolic effect. Collectively, these data suggest that PTH’s anabolic actions require coordinated signaling between bone and adipose, wherein a lipolytic response liberates fatty acids that are oxidized by osteoblasts to fuel bone formation.

## Graphical abstract

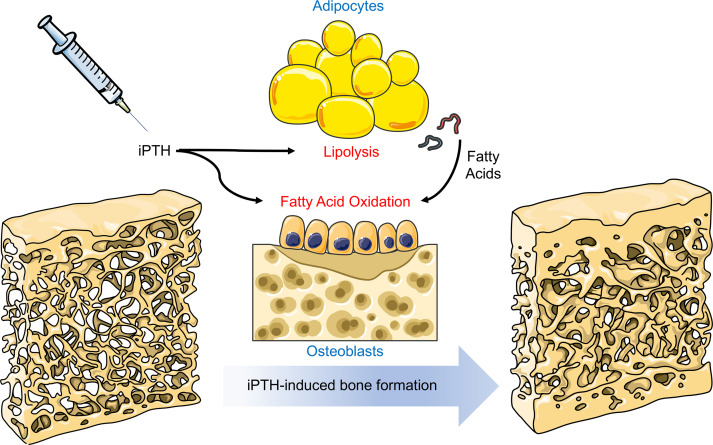

## Introduction

Parathyroid hormone (PTH) is an essential regulator of serum ionized calcium homeostasis. Deviations in calcium below the homeostatic range stimulate the release of the 84–amino acid peptide from the parathyroid gland, which then acts on the G protein–coupled PTH receptor (PTH1R) in target tissues. (1). In the kidney, PTH/PTH1R signaling stimulates calcium reabsorption and the production of vitamin D, which in turn stimulates calcium absorption in the small intestine. In the bone microenvironment, signaling indirectly stimulates bone resorption and the release of calcium stored in the bone matrix by increasing the expression of receptor activator of NF-κB ligand (RANKL), a key inducer of osteoclastogenesis, by osteoblasts (2). These actions restore serum calcium to the normal set point and lower serum PTH, but if PTH levels remain chronically high, as in the human condition of primary hyperparathyroidism, pathological bone loss ensues (3, 4).

When delivered intermittently (i.e., once daily) and at a low dose, PTH or a recombinant peptide containing amino acids 1–34 (PTH_1–34_, teriparatide) produces an anabolic response in bone in both preclinical animal models and humans (5, 6). Studies in rodents indicate that intermittent PTH (iPTH) increases osteoblast numbers, reduces osteoblast and osteocyte apoptosis, stimulates osteoprogenitor recruitment, and reactivates quiescent bone-lining cells to produce an increase in the rate of bone formation (7–10). As a result of its capacity to increase bone mineral density and reduce fracture risk in postmenopausal women, iPTH formed the basis for the first anabolic therapy for the treatment of osteoporosis (6, 11).

Engagement of PTH1R by PTH activates a complex network of intracellular signaling events within osteoblasts and osteocytes. Interaction of PTH1R with Gα_s_ stimulates the activation of adenylate cyclase and the production of cAMP, which is of prime importance for the overall anabolic response to iPTH in vivo (12, 13). In addition, PTH1R initiates phospholipase C and phospholipase D activity through an interaction with G_q_/G_11_ and G_12_/G_13_ (14, 15), respectively, while coupling with components of the osteoanabolic Wnt/β-catenin, insulin-like growth factor, and TGF-β pathways (16–18). However, much remains to be learned about how these pathways are integrated and lead to an increase in bone formation.

A relatively understudied contributor to the effects of PTH on osteoblast function and bone formation is the effect of the hormone on cellular metabolism. In early studies using undifferentiated osteoblasts, Neuman and colleagues (19–21) reported that PTH was a strong inducer of lactate production, which at the time was thought to be involved in bone resorption. Esen and colleagues (22) observed a similar effect in more recent studies using primary mouse osteoblasts and the MC3t3-E1 cell line. Intriguingly, this study also reported that despite a decrease in glucose oxidation, PTH treatment induced an increase in the rate of oxygen consumption, suggesting that the oxidation of another substrate is also under the control of PTH. Here, we explored the contribution of mitochondrial long-chain fatty acid β-oxidation, which yields significantly more ATP than glucose metabolism, to the anabolic effects of iPTH on bone. Adamek and colleagues (23) reported that PTH increases palmitate oxidation in specific bone cell populations while Catherwood (24) found that lipids support the proliferative response of the osteoblastic ROS17/2.8 cell line to PTH, but in vivo studies have not previously been completed.

After import into the cytoplasm and activation by acyl-CoA ligases, the metabolism of long-chain fatty acids is regulated by the sequential actions of the acyltransferases carnitine palmitoyl transferases 1 and 2 (CPT1a, CPT1b, or CPT1c and CPT2). The CPT1 isoform replaces CoA with carnitine, which allows the fatty acid to be recognized by carnitine-acylcarnitine translocase and transported into the mitochondrial matrix. CPT2 reverses the actions of CPT1 and thereby allows the initiation of the 4-step β-oxidation cycle that chain-shortens fatty acids and yields acetyl-CoA, NADH, and FADH_2_ for use in ATP generation. We reported previously that genetic disruptions in this pathway via the genetic ablation of *Cpt2*, which was selected because it is encoded by a single gene, leads to a low bone mass phenotype due to impaired osteoblast function (25).

In this work, we utilized genetic mouse models to demonstrate an essential role for mitochondrial long-chain fatty acid β-oxidation in the response to iPTH as mice deficient for *Cpt2* in osteoblasts are not able to form bone in response to a 6-week treatment regime. Interestingly, we also show that PTH induces adipose tissue lipolysis and that mice lacking the PTH1R or adipose triglyceride lipase specifically in adipocytes are resistant to PTH-induced anabolism. These data highlight a vital interaction between bone and adipose tissue wherein fatty acids liberated from adipocytes fuel the bone formation in response to iPTH.

## Results

### PTH enhances fatty acid oxidation by osteoblasts.

**Figure 1:**
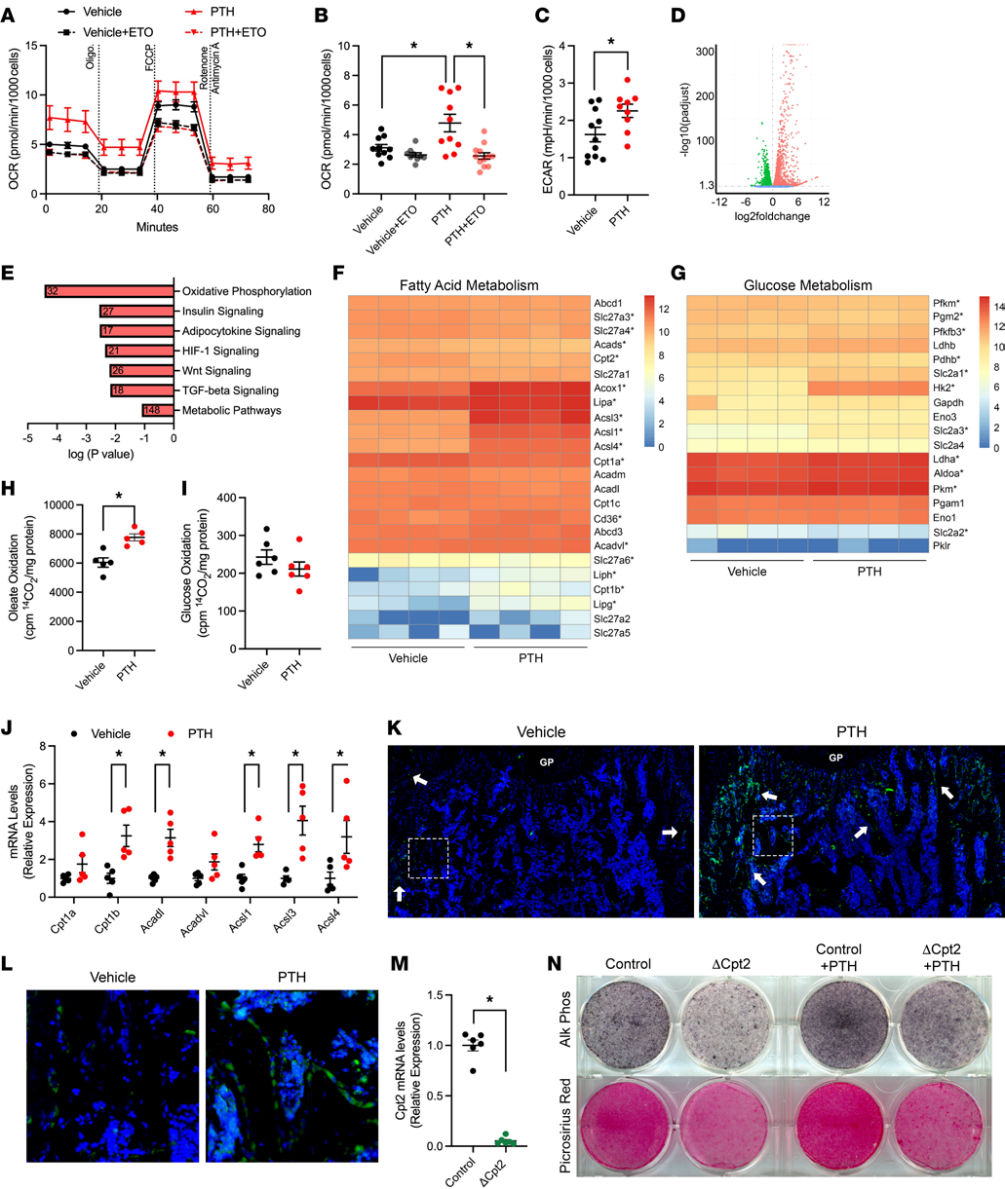
PTH influences osteoblast metabolism in vitro. (**A**) Mitochondrial stress tests quantifying oxygen consumption rate (OCR) by differentiating bone marrow stromal cells treated with vehicle or PTH for 2 hours with/without etomoxir (ETO), which was added 60 minutes before analysis. Oligomycin, FCCP, and rotenone/antimycin A were injected where shown by dashed lines. (**B** and **C**) OCR and extracellular acidification rate (ECAR) under basal conditions. (**D**–**G**) RNA sequencing was performed on differentiating osteoblast cultures treated with PTH for 24 hours. Volcano plot (**D**), gene enrichment for upregulated genes (**E**, numbers indicate the number of genes for each pathway), and heatmaps for genes associated with fatty acid (**F**) and glucose metabolism (**G**). For heatmaps, expression levels are shown using log_2_-transformed counts per million normalized values. Asterisks represent genes with an adjusted *P* value less than 0.05. (**H** and **I**) Oxidation of oleate and glucose in osteoblast cultures. (**J**) Quantitative PCR (qPCR) analysis of fatty acid oxidation genes in the femurs of C57BL/6 mice 4 hours after treatment with vehicle or PTH (100 μg/kg BW, *n* = 5 mice/treatment). (**K** and **L**) Assessment of fatty acid uptake in the femurs assessed with bodipy-palmitate. Mice were injected with vehicle or PTH 1 hour prior to injection of bodipy-palmitate and labeling for 3 hours. (**K**) Micrographs represent composite images of a 3 × 2 matrix of 20× original magnification fields of view. Arrows denote positively labeled osteoblasts/bone-lining cells. Results are representative of 3 mice per treatment group. GP, growth plate. (**L**) Enlarged images of boxed regions from **K**. (**M**) qPCR analysis of Cpt2 mRNA. (**N**) Osteoblast differentiation was assessed by alkaline phosphatase and collagen staining after treatment with saline control or PTH for 7 days. All data are represented as mean ± SEM. Data were analyzed by ANOVA with Tukey’s multiple comparisons post hoc test (**B**) or unpaired Student’s *t* test (all other panels). * *P* < 0.05.

To characterize the effect of PTH_1–34_ (hereafter PTH) on the intermediary metabolism of osteoblasts, we examined the hormone’s effects on cellular bioenergetics in cultures of differentiating mouse bone marrow stromal cells isolated from long bones as well as its effect on metabolic gene expression in cultures of calvarial osteoblasts. In stromal cell cultures, PTH rapidly (within 2 hours of treatment) increased oxygen consumption rate (OCR, Figure 1, A and B) and extracellular acidification rate (ECAR, Figure 1C). Since previous studies (22) indicated the effect on extracellular acidification is due to an increase in glycolytic metabolism in response to PTH, we predicted that the concomitant increase in oxygen consumption is due to enhanced mitochondrial long-chain fatty acid β-oxidation. Consistent with this idea, pretreating stromal cells with etomoxir (10 μM, 60 minutes prior to study), an irreversible inhibitor of CPT1 (26), abolished the increase in OCR after PTH treatment (Figure 1, A and B). In calvarial osteoblast cultures, RNA sequencing revealed that over 7,000 genes were differentially expressed after 24 hours of PTH treatment (*P*_adj_ < 0.05, Figure 1D). Of the 3,902 genes that were upregulated, Kyoto Encyclopedia of Genes and Genomes pathway analysis identified several expected PTH-responsive pathways, including Wnt signaling, TGF-β signaling, and HIF-1 signaling (Figure 1E). With regard to metabolic function, there was an enrichment of genes involved in the oxidative phosphorylation, the insulin signaling, and the adipocytokine signaling pathways. The adipocytokine signaling pathway included upregulated genes involved in fatty acid metabolism (Figure 1F), while the insulin signaling pathway included genes involved in glucose metabolism (Figure 1G). Of note, expression of genes encoding the fatty acid translocase Cd36 and acyl-CoA synthetases (Acsl1, Acsl3, Acsl4), which activate long-chain fatty acids for catabolism, as well as the rate-limiting enzyme Cpt1b, was upregulated by PTH treatment (Figure 1F). Expression of Hk2 and Slc2a1, involved in glucose utilization, was also upregulated as previously described (22). Upregulated genes related to oxidative phosphorylation included subunits of the NADH dehydrogenase, cytochrome *c* oxidase, and mitochondrial ATP synthase complexes essential to ATP generation.

To verify these findings, we first directly examined the ability of PTH to alter the metabolism of fatty acids and glucose by labeling calvarial osteoblasts with [1-^14^C]-oleic acid or [1-^14^C]-glucose. PTH induced a 28.5% increase in the oxidation of oleate to CO_2_ (Figure 1H) but had no effect on glucose oxidation (Figure 1I). Second, we treated C57BL/6 mice with vehicle or PTH (100 μg/kg BW) for 4 hours and examined gene expression in the femur. Similar to our in vitro studies, PTH increased the expression of genes involved in fatty acid oxidation, including Cpt1b, Acadl, and 3 acyl-CoA synthase isoforms (Figure 1J). Next, we examined fatty acid uptake by treating mice with vehicle or PTH prior to labeling with palmitate-bodipy. Limited uptake of the labeled fatty acid was evident in the distal femurs of vehicle-treated mice, but there was a striking enrichment in osteoblasts and bone-lining cells in those treated with PTH (Figure 1, K and L). Finally, we treated calvarial osteoblasts expressing or deficient for the *Cpt2* gene (ΔCpt2, Figure 1M), encoding Cpt2, with PTH as they differentiated. As in our previous work (25), ΔCpt2 osteoblasts exhibited an impairment in osteoblast differentiation when assessed by staining for alkaline phosphatase activity or Picrosirius red staining for collagen deposition (Figure 1N). Daily PTH treatment markedly increased markers of osteoblast differentiation in control osteoblasts but produced only a moderate response in ΔCpt2 osteoblasts, with the intensity of staining remaining lower than that of untreated control osteoblast cultures. Taken together these data indicated that PTH induces robust changes in osteoblastic metabolism, including an enhancement in fatty acid uptake and oxidation that appears to be required for the hormone’s effect on osteoblast function.

### Osteoblastic Cpt2 deficiency abolishes iPTH-induced bone formation.

**Figure 2:**
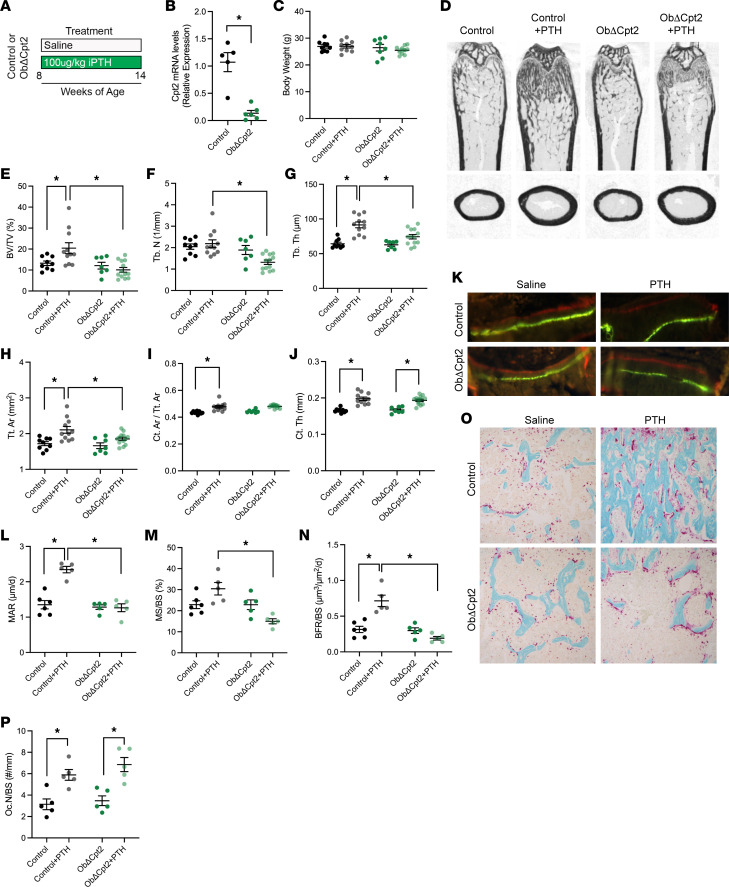
Fatty acid oxidation is required for iPTH-induced anabolism. (**A**) Depiction of treatment scheme used for control and ObΔCpt2 mice as well as mice used in experiments described later. (**B**) qPCR analysis of Cpt2 mRNA levels in the femurs of control and ObΔCpt2 mice (*n* = 7 mice/genotype). (**C**) Body weights at the conclusion of 6 weeks of vehicle or PTH treatment (*n* = 8–10 mice/genotype). (**D**) Representative microCT images for the distal femur and the femoral mid-diaphysis. (**E**–**G**) Quantification of trabecular bone volume per tissue volume (BV/TV, **E**), trabecular number (Tb. N, **F**) and trabecular thickness (Tb. Th, **G**) in the distal femur (*n* = 7–13 mice/genotype). (**H**–**J**) Quantification of cortical tissue area (Tt. Ar, **H**), cortical bone area per tissue area (Ct. Ar/Tt. Ar, **I**), and cortical thickness (Ct. Th, **J**) at the femoral mid-diaphysis (*n* = 7–13 mice/genotype). (**K**) Representative micrographs of calcein and alizarin red incorporation in the trabecular bone compartment used to calculate dynamic indices of bone formation. Original magnification, 40×. (**L**–**N**) Quantification of mineral apposition rate (MAR, **L**), mineralizing surface per bone surface (MS/BS, **M**) and bone formation rate per bone surface (BFR/BS, **N**) in the trabecular bone compartment of distal femurs (*n* = 5–6 mice/genotype). (**O**) Representative micrographs after tartrate-resistant acid phosphatase staining for osteoclasts. Original magnification, 10×. (**P**) Quantification of osteoclast number per bone surface (Oc.N/BS, *n* = 5 mice/genotype). All data are represented as mean ± SEM. Data were analyzed by unpaired Student’s *t* test (**A**) or ANOVA with Tukey’s multiple comparisons post hoc test (all other panels). * *P* < 0.05.

As a counterpart to the in vitro osteoblast differentiation studies and to determine if mitochondrial long-chain fatty acid β-oxidation is required for iPTH administration to induce bone formation, 8-week-old mice lacking *Cpt2* specifically in osteoblasts and osteocytes (Cpt2^fl/fl^ Ocn-Cre^Tg/+^, hereafter ObΔCpt2) and control littermates (Cpt2^fl/fl^) were treated with PTH (100 μg/kg body weight) 5 days per week for 6 weeks (Figure 2, A and B). We targeted *Cpt2* as it is encoded by a single gene (3 Cpt1 genes are present in mammals) and completed these studies in male mice as our previous work demonstrated that male ObΔCpt2 have normal bone structure at this age (25), and as a result there should be no difference in the baseline phenotype at the initiation of the study. After the 6-week treatment period, body weights were comparable in all groups (Figure 2C), but micro-computed tomography (microCT) imaging of the femurs revealed distinct differences in the response to iPTH (Figure 2, D–J). In control mice, iPTH treatment induced a 53.9% increase in trabecular bone volume per tissue volume in the distal femur relative to saline-treated littermates (Figure 2E) that was secondary to a 23.0% increase in trabecular thickness (Figure 2G). By contrast, iPTH had no effect on trabecular volume in ObΔCpt2 mice as trabecular thickness was not increased, and trabecular number exhibited a downward trend relative to saline-treated ObΔCpt2 mice (*P* = 0.067) and was significantly reduced relative to iPTH-treated control mice (Figure 2F). Similar reductions in the anabolic response to iPTH in ObΔCpt2 mice were also evident in the cortical bone compartment at the femoral mid-diaphysis (Figure 2, D and H–J). Whereas control iPTH-treated mice exhibited a 22.6% increase in tissue area relative to saline-treated controls (Figure 2H), tissue area was increased by just 11.6% in ObΔCpt2 iPTH-treated mice relative to saline-treated ObΔCpt2 mice.

To determine the cellular basis for the reductions in the anabolic response to iPTH in ObΔCpt2 mice, we performed dynamic histomorphometric analyses of osteoblast function and static analyses of osteoclast numbers in the trabecular bone compartment of the distal femur where the difference between control and ObΔCpt2 mice was most pronounced. In control mice, iPTH stimulated a marked increase in the mineral apposition rate (Figure 2, K and L), which in combination with a trend toward an increase in mineralizing surface per bone surface (*P* = 0.09, Figure 2M) led to a 127.1% increase in the bone formation rate per bone surface (Figure 2N) as expected. However, these indices of osteoblastic function were unaffected by iPTH in ObΔCpt2 mice. Similar to the effect on trabecular bone structure, the mineral apposition rate was not increased, and the mineralizing surface trended downward in iPTH-treated ObΔCpt2 mice, which led to the overall bone formation rate not being affected by iPTH in the mutant mice. Though not directly examined, the changes in tissue geometry imply a similar defect in the cortical bone compartment. Since appositional growth on the periosteal surface is carried out by osteoblasts, the diminished increase in tissue area in ObΔCpt2 mice (Figure 2H) implies a defect in osteoblastic activity. The identical increase in cortical bone thickness in control and ObΔCpt2 after iPTH treatment (Figure 2J) suggests that the mutant mice have deposited new bone on the endosteal surface or exhibited a decrease in endosteal resorption. Assessment of osteoclast numbers in the distal femur after tartrate-resistant acid phosphatase staining revealed that iPTH increased osteoclastogenesis to a similar degree in control and ObΔCpt2 mice (Figure 2, O and P). These data are complementary to the impaired differentiation of ΔCpt2 osteoblasts treated with PTH in vitro and indicate that the catabolism of long-chain fatty acids is essential for osteoblasts to form new bone in response to iPTH but not for their ability to signal to osteoclasts.

### PTH alters whole-body lipid homeostasis.

**Figure 3:**
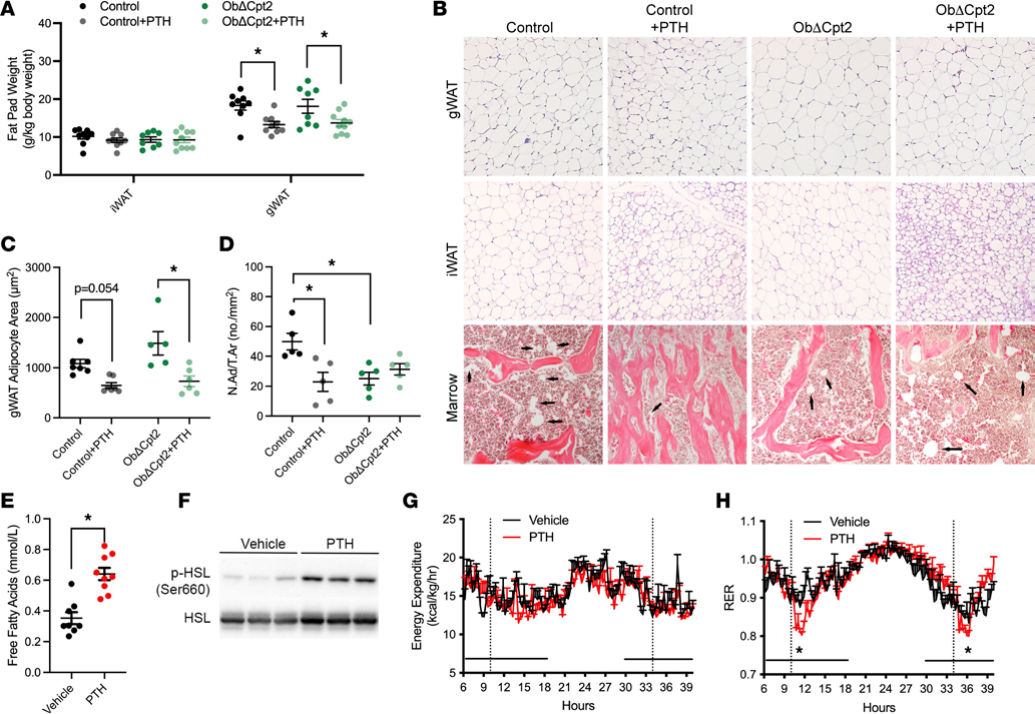
iPTH alters adipocyte morphology and increases serum free fatty acids. (**A**) Inguinal (iWAT) and gonadal (gWAT) fat pad weights in control and ObΔCpt2 mice after 6 weeks of iPTH or vehicle treatment (*n* = 8–10 mice/genotype). (**B**) Representative micrographs of hematoxylin and eosin–stained sections of gWAT, iWAT, and bone marrow in the distal femurs. Original magnification, 10×. (**C**) Quantification of gWAT adipocyte size area (*n* = 5–7 mice/genotype). (**D**) Adipocyte numbers per tissue area in the distal femurs (N.Ad/T.Ar, *n* = 5 mice/genotype). (**E**) Serum free fatty acids in C57BL/6 mice 30 minutes after being treated with vehicle or PTH (*n* = 8–9 mice/treatment). (**F**) Representative Western blot of hormone-sensitive lipase (HSL) phosphorylation in iWAT 30 minutes after vehicle or PTH treatment. (**G** and **H**) Indirect calorimetric assessment of energy expenditure (**G**) and respiratory exchange ratio (**H**) in C57BL/6 mice treated with vehicle or PTH where shown by the vertical dotted lines (*n* = 8 mice/treatment). All data are represented as mean ± SEM. Data were analyzed by unpaired Student’s *t* test (**E** and **G**) or ANOVA with Tukey’s multiple comparisons post hoc test (all other panels). * *P* < 0.05.

Having established long-chain fatty acids as an essential fuel source for iPTH-induced bone formation, we next questioned the primary source of fatty acids for this response. Since intravenous administration of parathyroid extracts in humans produces a transient increase in serum free fatty acids (27), we hypothesized that PTH induces a lipolytic response in adipose that liberates fatty acids to be used by osteoblasts. The first indication of this interaction was found in the analysis of body composition and adipocyte morphometry in control and ObΔCpt2 mice. iPTH treatment significantly decreased the wet weight of the gonadal white adipose tissue (gWAT) depot in both control and mutant mice (Figure 3A) as well as the size of adipocytes in histological sections (Figure 3, B and C). Wet weight of the inguinal adipose depot (iWAT) was not affected by iPTH in either genotype, but adipocyte size was reduced and accompanied by a dramatic increase in the abundance of multilocular adipocytes in iPTH-treated control and ObΔCpt2 mice (Figure 3B). A decrease in adipocyte numbers per tissue area was also evident in the marrow of control mice treated with iPTH (Figure 3, B and D) but not in ObΔCpt2 mice that already exhibited a paucity of marrow adipocytes.

We tested our hypothesis more directly by examining acute effects of PTH on lipid homeostasis in C57BL/6J mice. Similar to the effect in humans (27), PTH treatment significantly increased serum free fatty acids (30 minutes posttreatment, Figure 3E) and induced the activating phosphorylation of hormone-sensitive lipase in white adipose (Figure 3F). Additionally, when global energy balance was examined by indirect calorimetry (Figure 3, G and H), PTH treatment induced a rapid reduction in the respiratory exchange ratio, indicative of increased lipid catabolism, without affecting overall energy expenditure. Thus, iPTH administration reduces fat mass while inducing lipolysis and rapid, systemic changes in lipid homeostasis that may be used to fuel bone formation.

### Pth1r expression by adipocytes is required for iPTH-induced bone formation.

**Figure 4:**
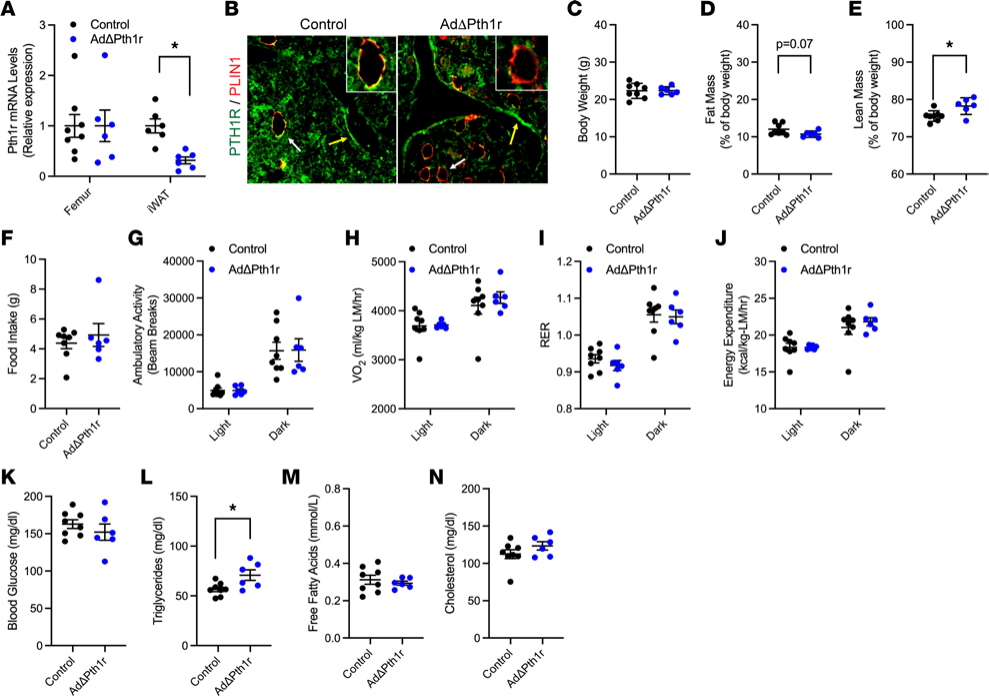
Pth1r ablation in adipocytes has a modest effect on body composition. (**A**) qPCR analysis of *Pth1r* mRNA levels in the femurs and iWAT of control and AdΔPth1r mice (*n* = 6–8 mice/genotype). (**B**) Representative micrographs of PTH1R and PLIN1 co-immunostaining in the distal femur of control and AdΔPth1r mice. Note lack of costaining in marrow PLIN1^+^ adipocytes in AdΔPth1r mice (20× original magnification). White arrows indicate adipocytes; yellow arrows indicate bone-lining cells. (**C**–**E**) Body composition, including body weight (**C**) as well as fat (**D**) and lean body mass (**E**), was assessed at 8 weeks of age in male control and AdΔPth1r mice (*n* = 6–8 mice/genotype). (**F**) Food intake at 8 weeks of age (*n* = 6–8 mice per genotype). (**G**) Ambulatory activity assessed by beam breaks in 12-hour light and dark periods (*n* = 6–8 mice/genotype). (**H**–**J**) Indirect calorimetry measures of energy expenditure (*n* = 6–8 mice/genotype). LM, lean mass. (**K**–**N**) Serum metabolites in random fed control and AdΔPth1r mice (*n* = 6–8 mice/genotype). All data are represented as mean ± SEM. Data were analyzed by unpaired Student’s *t* test. * *P* < 0.05.

PTH exerts its effects by signaling through the G protein–coupled receptor PTH1R that is highly expressed in bone, kidney, and intestine (28). As a test of whether PTH1R signaling in adipocytes is required for the lipolytic response and for the bone formation response to PTH, we generated mice in which *Pth1r* is ablated specifically in adipocytes (Pth1r^fl/fl^ AdipoQ-Cre^Tg/+^, hereafter AdΔPth1r) and control littermates (Pth1r^fl/fl^) (29). The specificity of *Pth1r* gene deletion was confirmed by qPCR analysis and immunostaining. In AdΔPth1r mice, Pth1r expression was reduced by 68.4% in white adipose tissue relative to controls but normally expressed in the femur (Figure 4A). Similarly, co-immunostaining for PTH1R and perilipin-1 (PLIN1) in the distal femur demonstrated that PTH1R expression was lost in marrow adipocytes (Figure 4B) but present in bone-lining cells, osteoblasts, and other marrow components in the mutant mice.

When examined at 8 weeks of age, body weight was comparable in control and AdΔPth1r mice (Figure 4C), but quantitative NMR (qNMR) analysis of body composition revealed subtle changes in body composition. Relative to controls, AdΔPth1r mice exhibited a trend toward reduced fat mass (Figure 4D, *P* = 0.07) and a small, but statistically significant, increase in lean mass (Figure 4E). In line with this modest phenotype, food intake, activity levels, and measures of energy expenditure were similar in the control and mutant mice when assessed by indirect calorimetry (Figure 4, F–J). Likewise, serum triglycerides were elevated in AdΔPth1r mice, but all other serum metabolites were normal (Figure 4, K–N).

**Figure 5:**
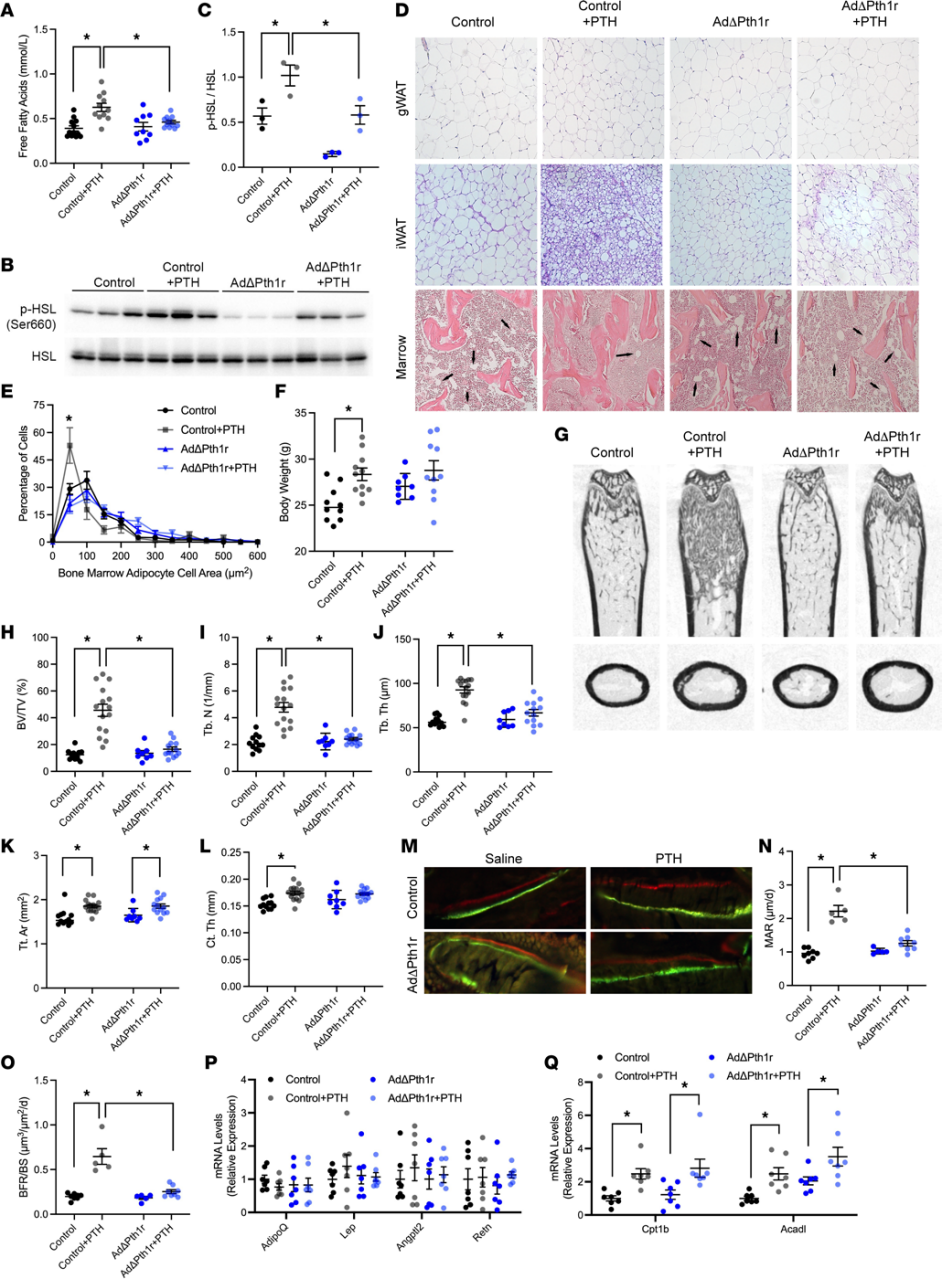
iPTH signals in adipose tissue to regulate bone formation. (**A**) Serum free fatty acids in control and AdΔPth1r mice 30 minutes after being treated with vehicle or PTH (*n* = 9–12 mice/genotype). (**B** and **C**) Representative Western blot and quantification of HSL phosphorylation in iWAT 30 minutes after vehicle or PTH treatment with quantification (*n* = 3 mice/genotype). (**D**) Representative micrographs of hematoxylin and eosin–stained sections of gWAT, iWAT, and bone marrow in the distal femurs (10× original magnification). Arrows indicate marrow adipocytes. (**E**) Quantification of bone marrow adipocyte size (*n* = 6 mice/genotype). (**F**) Body weights at the conclusion of 6 weeks of vehicle or PTH treatment (*n* = 8–11 mice/genotype). (**G**) Representative microCT images for the distal femur and the femoral mid-diaphysis. (**H**–**J**) Quantification of trabecular bone volume per tissue volume (BV/TV, **H**), trabecular number (Tb. N, **I**), and trabecular thickness (Tb. Th, **J**) in the distal femur (*n* = 8–15 mice/genotype). (**K**–**M**) Quantification of cortical tissue area (Tt. Ar, **K**) and cortical thickness (Ct. Th, **L**) at the femoral mid-diaphysis (*n* = 8–15 mice/genotype). (**M**) Representative micrographs of calcein and alizarin red incorporation in the trabecular bone compartment used to calculate dynamic indices of bone formation. Original magnification, 40×. (**N** and **O**) Quantification of mineral apposition rate (MAR, **N**), and bone formation rate per bone surface (BFR/BS, **O**) in the trabecular bone compartment of distal femurs (*n* = 5–8 mice/genotype). (**P**) qPCR analysis of adipokine expression in iWAT (*n* = 7 mice/genotype). (**Q**) qPCR analysis of fatty acid oxidation genes in femurs (*n* = 7 mice/genotype). All data are represented as mean ± SEM. Data were analyzed by unpaired Student’s *t* test (**E**) or ANOVA with Tukey’s multiple comparisons post hoc test (all other panels). * *P* < 0.05.

Disrupting *Pth1r* expression in adipocytes largely abolished the acute lipolytic response to PTH administration as serum fatty acids were not increased by the hormone in AdΔPth1r mice (Figure 5A), and the increase in HSL phosphorylation after PTH treatment was greatly diminished relative to that in treated control mice (Figure 5, B and C). The increase in HSL phosphorylation observed in AdΔPth1r mice relative to saline-treated mutants is likely to be due to other cell types present in fat pads that are not targeted by the AdipoQ-Cre transgene. The change in adipocyte morphology after 6 weeks of iPTH treatment was also inhibited in AdΔPth1r mice. Whereas adipocyte size and abundance were reduced in the gWAT, iWAT, and marrow of the distal femur in control iPTH-treated mice relative to untreated controls, morphology and abundance in iPTH-treated mutants were nearly identical to saline-treated mutants (Figure 5, D and E).

Control mice (Pth1r^fl/fl^) exhibited a 12.8% increase in body weight after 6 weeks of iPTH treatment that was not evident in AdΔPth1r mice (Figure 5F) and could be related to the robust bone formation evident in this strain (Figure 5G). Comparison of the anabolic response to iPTH between control and AdΔPth1r mice revealed a phenotype in the distal femur that was nearly identical to that observed in ObΔCpt2 mice. iPTH increased bone volume per tissue volume secondary to increases in trabecular number and trabecular thickness in control mice, but these parameters of trabecular bone structure were not affected by iPTH in AdΔPth1r mice (Figure 5, G–J). Likewise, iPTH increased the mineral apposition rate, which led to an increase in the bone formation rate, in the trabecular bone compartment of control mice that were not evident in AdΔPth1r mice (Figure 5, M–O). Interestingly, both control and AdΔPth1r mice exhibited increases in tissue area in response to iPTH treatment (Figure 5, G, K, and L). Since cortical appositional growth was inhibited in ObΔCpt2 mice treated with iPTH, these data imply that periosteal osteoblasts are able to extract sufficient fatty acids for growth from the circulation and do not require concomitant actions in adipose tissue. Importantly, the expression of genes encoding adipokines, including *Adipoq*, *Lep*, *Angptl2*, and *Retn*, was not affected by PTH treatment or the ablation of the *Pth1r* in adipocytes (Figure 5P). Together, these data indicate a requirement for PTH signaling in bone as well as in adipose to orchestrate an increase in trabecular bone volume. Since the lipolytic response is impaired (Figure 5A), but PTH still induces oxidative gene expression in bone (Figure 5Q), the primary defect would appear to be a lack of sufficient energy supply.

### Adipose tissue lipolysis is required for iPTH-induced bone formation.

**Figure 6:**
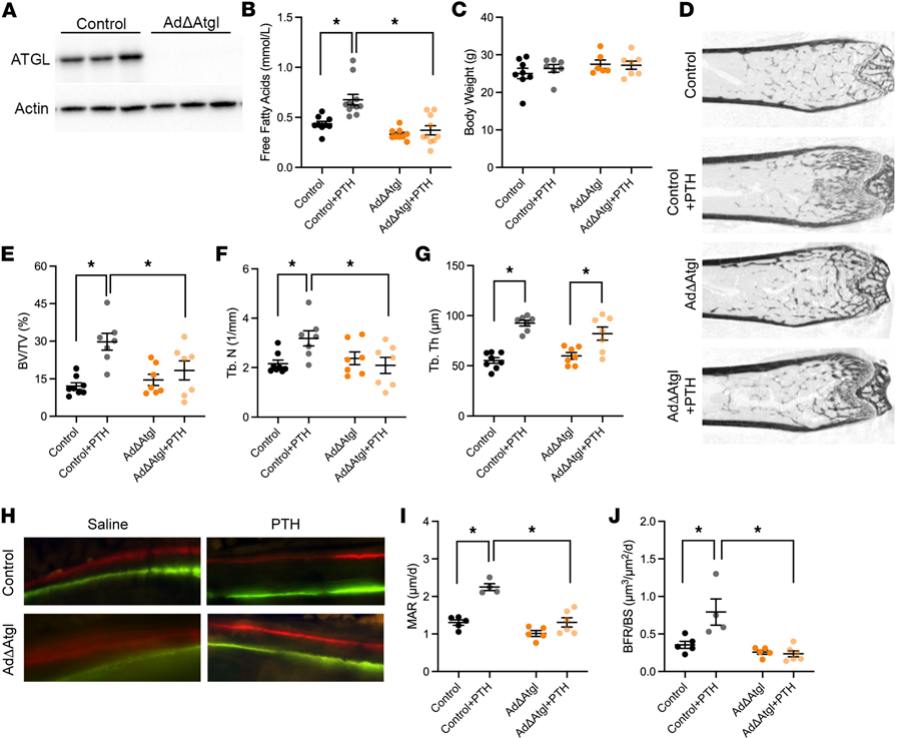
AdΔAtgl mice have a diminished response to iPTH. (**A**) Representative Western blot of ATGL protein in iWAT of control and AdΔAtgl mice. (**B**) Serum free fatty acids in control and AdΔAtgl mice 30 minutes after being treated with vehicle or PTH (*n* = 9–11 mice/genotype). (**C**) Body weights at the conclusion of 6 weeks of vehicle or PTH treatment (*n* = 6–8 mice/genotype). (**D**) Representative microCT images for the distal femurs. (**E**–**G**) Quantification of trabecular bone volume per tissue volume (BV/TV, **E**), trabecular number (Tb. N, **F**), and trabecular thickness (Tb. Th, **G**) in the distal femur (*n* = 7–8 mice/genotype). (**H**) Representative micrographs of calcein and alizarin red incorporation in the trabecular bone compartment used to calculate dynamic indices of bone formation. Original magnification, 40×. (**I** and **J**) Quantification of mineral apposition rate (MAR, **I**) and bone formation rate per bone surface (BFR/BS, **J**) in the trabecular bone compartment of distal femur (*n* = 4–6 mice/genotype). All data are represented as mean ± SEM. Data were analyzed by ANOVA with Tukey’s multiple comparisons post hoc test. * *P* < 0.05.

In a final series of experiments, we treated mice lacking adipose triglyceride lipase (ATGL) in adipocytes (Pnpla2^fl/fl^ AdipoQ-Cre^Tg/+^, hereafter AdΔAtgl; Figure 6A) and control littermates (Pnpla2^fl/fl^) (30–32) with PTH to determine if lipolysis downstream of PTH1R activation in adipocytes is necessary for the bone formation response. As expected, the increase in serum free fatty acids after acute PTH treatment evident in control mice was eliminated in AdΔAtgl mice (Figure 6B), which lack the first and rate-limiting enzyme in lipolysis. iPTH treatment for 6 weeks did not affect body weight in either control or AdΔAtgl mice (Figure 6C), but the anabolic response to iPTH was diminished in AdΔAtgl mice (Figure 6, D–G). In control mice, trabecular bone volume per tissue volume was increased by 125.0% by iPTH treatment, due to significant increases in trabecular number and trabecular thickness, but was not significantly changed in AdΔAtgl mice. Further, iPTH increased both the mineral apposition rate and the bone formation rate per bone surface in control mice, but AdΔAtgl mice were unresponsive (Figure 6, H–J). Together, these genetic data highlight a potentially novel interaction between bone and adipose wherein the liberation of fatty acids from adipocytes in response to PTH fuels the anabolic response by osteoblasts.

## Discussion

In these studies, we used cell culture models and genetic mouse models to examine the metabolic requirements of the osteoblast in response to a clinically relevant osteoanabolic signal, iPTH. We focused on mitochondrial long-chain fatty acid β-oxidation because in vitro studies using differentiating bone marrow stromal cells and calvarial osteoblasts revealed acute changes in fatty acid catabolism, as evidenced by the etomoxir-sensitive increase in oxygen consumption, as well as longer term changes in the expression of genes encoding enzymes necessary for fatty acid acquisition and oxidation. Moreover, our previous work (25) demonstrated a requirement for mitochondrial long-chain fatty acid β-oxidation for normal osteoblastic activity particularly in young mice, when bone mass is rapidly being accumulated and growth-promoting signals are abundant.

We demonstrated here that fatty acid catabolism is essential for the anabolic response to PTH. Osteoblasts deficient for *Cpt2*, which encodes an obligate enzyme in fatty acid oxidation, exhibited an impairment in matrix mineralization in vitro when treated with PTH while ObΔCpt2 mice were almost completely resistant to the anabolic actions of iPTH in vivo. These data are compatible with the aforementioned findings from Adamek (23) and Catherwood (24) wherein PTH increased fatty acid oxidation by specific osteoblast populations and lipid supplementation augmented the effect of PTH on osteoblast proliferation, respectively. The histomorphometric studies performed in control and ObΔCpt2 mice indicated that the defect in the mutant mice was due to an inability of iPTH to increase the rate of osteoblastic activity when β-oxidation is impaired. It is clear that the defect is not due to a generalized defect in PTH sensitivity since the hormone increased osteoclastogenesis, a process that proceeds via the induction of RANKL expression by osteoblasts (2), in both control and ObΔCpt2 mice. Instead, we suspect the inhibition of osteoblastic activity is due to energetic deficiencies like those found in ΔCpt2 osteoblasts treated with estrogen (25).

PTH treatment also increased glucose utilization as demonstrated by the increase in ECAR in bone marrow stromal cell cultures and the upregulation of genes encoding GLUT1, hexokinase-2, and lactate dehydrogenase. This response was observed previously by Esen and colleagues (22), who reported that PTH stimulates glycolytic metabolism but downregulates oxidative metabolism of glucose in osteoblasts. Further, glucose utilization is known to be vital for full osteoblast function since mice rendered deficient for GLUT1 in early osteoblasts exhibit severe defects in skeletogenesis (33), so this effect of PTH was expected. The β-oxidation of fatty acids has greater energy-generating potential than glycolytic metabolism of glucose. However, it is not possible to know at this point if one pathway is dominant to the other, even though the defect reported here in ObΔCpt2 mice appears to be greater than when iPTH-treated mice are also treated with dichloroacetate to increase glucose oxidation at the expense of glycolysis (22). These studies would require a head-to-head comparison of the PTH response in the Cpt2 mutants used here and mice lacking lactate dehydrogenase or another enzyme in glycolysis in the same population of osteoblasts (i.e., Ocn-Cre^Tg/+^). Glucose uptake and lactate production are increased in osteoblasts lacking Cpt2 (25), but the effect of inhibiting glucose metabolism on fatty acid oxidation in osteoblasts has not been examined to our knowledge. Utilization of glutamine, another potential energy source, in response to PTH is primarily used for synthetic purposes and to maintain redox homeostasis (34).

Our studies further suggest that the trabecular osteoblast’s need for fatty acids is supported by PTH signaling in adipocytes. Bone-adipose interactions have been studied in several contexts as a result of the increase in bone marrow adiposity as bone mass decreases during the aging process (35, 36) and the negative association between body mass index and bone mass (37–39). On the one hand, adipocytes produce adipokines, such as leptin and adiponectin, that suppress osteoblast function and lead to bone loss (40–42). This interaction may partially explain the robust bone formation that occurs in “fat-free” mice (43). On the other hand, the storage of triglycerides in mature adipocytes represents a potential energy supply for bone formation. Indeed, Li and colleagues (44) demonstrated a requirement for the release of fatty acids from bone marrow adipocytes to maintain bone mass in the face of energy deficits while Maridas and colleagues (45) reported that PTH stimulates the transfer of fatty acids from bone marrow adipocytes to bone marrow stromal cells in in vitro systems. The ability of PTH to stimulate lipolysis in adipose tissue, which in turn fuels bone formation, appears to be the main point of interaction in this context as we found no changes in the expression of adipokines in fat pads after PTH treatment, and bone formation after iPTH was dramatically impaired in AdΔAtgl mice.

Bone marrow adipocytes could represent a local source of fatty acids to be catabolized by osteoblasts. In accordance with this idea, 6 weeks of iPTH reduced the abundance of adipocytes in the bone marrow in control mice but not in mice where PTH1R was ablated. We suspect that this relates in part to the release of stored lipids in response to iPTH as well as the role of PTH/PTH1R signaling in osteoblast versus adipocyte lineage specification (46, 47). However, our data show that larger white adipose depots, such as the inguinal and gonadal fat pads, also supply fatty acids that osteoblasts can use. Evidence for this assertion include 1) iPTH produced a systemic change in lipid homeostasis marked by a reduction in the respiratory exchange ratio, 2) iPTH reduced the mass of the gonadal adipose depot and the size of adipocytes in both the gonadal and inguinal depots, and 3) iPTH stimulated the activating phosphorylation of HSL in fat pads. It is possible that bone marrow adipocytes are engaged first as the local source of fat, and other more distant depots become involved in accordance with the strength and duration of the anabolic signal. Additional studies would be necessary to determine if this is the case.

While both ObΔCpt2 mice and AdΔPth1R mice exhibited reduced trabecular bone formation in response to iPTH, only the ObΔCpt2 exhibited a defect in the effect of iPTH on cortical bone. These data suggest that long-chain fatty acid oxidation is required for iPTH to increase periosteal apposition (since cortical tissue area was not increased in ObΔCpt2 mice) but that signaling in adipose tissue is not required for this response. This could be due to differences in the ability of cortical osteoblasts and cancellous osteoblasts to extract lipid from the circulation, differences in the amount of fatty acids that must be oxidized by each osteoblast population to generate an anabolic response, or differences in the rates of bone modeling/remodeling in the 2 bone compartments.

In summary, these studies uncover details on the bioenergetics of the osteoblasts and the mechanisms by which iPTH induces bone formation. PTH must initiate signaling in the osteoblast as well as in the adipocyte to increase bone mass, with the latter providing fatty acids that can fuel the response of the osteoblast. It is possible that further examination of the interaction between bone and adipose in this context will lead to the treatment of metabolic bone disease by enhancing or extending the osteoanabolic actions of PTH.

## Methods

### Animal models.

Mice were housed on ventilated racks on a 14-hour light/10-hour dark cycle and fed ad libitum with a standard chow diet (Extruded Global Rodent Diet, Harlan Laboratories). Mice in which exon 4 of the mouse *Cpt2* gene is flanked by *loxP* sequences (48, 49) were crossed with Ocn-Cre^Tg/+^ (50) (both provided in-house) to generate osteoblast-specific knockouts as described previously (25). Pth1r^fl/fl^ mice (29) were provided by Henry Kronenberg (Massachusetts General Hospital, Boston, Massachusetts, USA) and crossed with AdipoQ-Cre^Tg/+^ mice (The Jackson Laboratory strain 028020) (51) to generate adipocyte-specific knockouts. Atgl^fl/fl^ mice (30, 31) were provided by Erin Kershaw (University of Pittsburgh, Pittsburgh, Pennsylvania, USA) and were also crossed with AdipoQ-Cre^Tg/+^ mice. All transgenic mice were maintained on a C57BL/6 background. C57BL/6J mice were obtained from The Jackson Laboratory. Genotypes were determined by qPCR analysis of genomic DNA isolated from tail biopsies.

### PTH injections.

For studying acute effects of PTH in vivo, 100 μg/kg PTH_1–34_ (BACHEM) resuspended in culture-grade PBS (Corning), PBS, or vehicle control was injected subcutaneously. Tissues and serum were collected 30 minutes after injection. For intermittent treatment, male mice were weighed and randomly assigned to the vehicle or iPTH group. Vehicle or iPTH (100 μg/kg PTH_1–34_) was injected 5 days per week for 6 weeks. Body weights were collected weekly and injection volumes adjusted accordingly.

### Fatty acid uptake in vivo.

Bodipy-palmitate (also known as Bodipy-C16, 1 mg/mL, Cayman Chemical) was conjugated to fatty acid–free bovine serum albumin (BSA, MilliporeSigma) in a reaction consisting of 1 volume of palmitate-bodipy to 8.3 volumes of 20% BSA in sterile saline. Conjugation was carried out by incubating the reaction mix at 37°C for 30 minutes. For uptake studies, 8-week-old male C57BL/6 mice were injected s.c. with PBS, vehicle control, or 100 g/kg PTH_1–34_ 1 hour prior to i.p. injection of bodipy-palmitate/BSA (50 μg/g BW final bodipy-palmitate concentration). Mice were euthanized 3 hours after injection, and hind limbs were fixed in 4% paraformaldehyde prior to cryosectioning. Sections were imaged at 20× original magnification with a Nikon Ti2-E widefield fluorescence microscope.

### Skeletal phenotyping.

High-resolution images of the mouse femur were acquired using a desktop microtomographic imaging system (Skyscan 1275, Bruker), in accordance with the recommendation of the American Society for Bone and Mineral Research (ASBMR) (52). Bones were scanned with an isotropic voxel size of 10 μm at 65 keV and 153 μA using a 1.0 mm aluminum filter. Trabecular bone parameters in the distal femur were assessed in a region of interest 500 μm proximal to the growth plate and extending for 2 mm (200 CT slices). Femoral cortical bone structure was assessed in a 500 μm region of interest centered on the mid-diaphysis.

Dynamic bone formation was assessed in the final week of iPTH administration by i.p. injection of calcein 10 days prior to sacrifice followed by i.p. injection of alizarin 3 days prior to sacrifice. After embedding femoral samples in methylmethacrylate, 5 μm sections were cut with a Leica microtome (RM2265). Dynamic histomorphometry indices were measured at standardized sites under the growth plate using a semiautomatic method (BIOQUANT OSTEO) in compliance with the guidelines established by the nomenclature committee from the ASBMR (53). For static measurements of osteoclast numbers and marrow adipocyte numbers, femurs were decalcified using 14% EDTA and paraffin-embedded. After embedding, 5 μm sections were cut and stained with H&E or stained for TRAP activity and analyzed using BIOQUANT OSTEO. Quantification was performed in a region of interest one field below the growth plate (to approximate the offset used in microCT analyses) in the center of the metaphysis. For immunofluorescence, decalcified femurs were embedded in OCT (Tissue Tek) and 10 μm sections were cut. Sections were stained for anti-PTH1R (MilliporeSigma 05-517) and anti-PLIN1 (Cell Signaling Technology 9349S) primary antibodies and appropriate secondary antibodies before imaging with a Nikon Eclipse Ci with Photofluor LM75 light source.

### Metabolic profiling and bioassays.

For indirect calorimetry studies, 8-week-old male mice were individually housed in a Comprehensive Laboratory Animal Monitoring System (Columbus Instruments). Calorimetry, daily body weight, and daily food intake data were acquired during a 4-day experimental period. Animals were allowed to acclimate for 2 days before saline or PTH_1–34_ (100 μg/kg) injections on day 3 and day 4. Oxygen consumption (VO_2_, mL/kg/h) and carbon dioxide production (VCO_2_) were measured for each chamber every 20 minutes throughout the study. Respiratory exchange ratio (RER = VCO_2_/VO_2_) was calculated by Oxymax software (v 4.90) to estimate relative oxidation of carbohydrates (RER = 1.0) compared with fat (RER approaching 0.7), not accounting for protein oxidation. Energy expenditure was calculated as VO_2_ × (3.815 + [1.232 × RER]) and normalized for lean body mass (kcal/kg/h) assessed by qNMR (Echo MRI). Plasma free fatty acids (MilliporeSigma), triglycerides (MilliporeSigma), and cholesterol (MilliporeSigma) were measured colorimetrically in plasma collected 30 minutes after administration of the final PTH injection. Mouse adipose tissue was fixed in 4% paraformaldehyde for 24 hours at 4°C and washed with PBS. Adipose tissue samples were paraffin-embedded and stained with H&E using standard methods. Adipocyte size was measured using ImageJ (NIH).

### Seahorse analysis.

Bone marrow stromal cells were isolated from femurs and tibia from 8-week-old C57BL/6 mice. Adherent cells were trypsinized and seeded in a 96-well cell Seahorse culture microplate (Agilent) at 2.5 × 10^4^ cells per well density. Cells were differentiated for 7 days in MEM containing 10% serum with 5 mM β-glycerol phosphate and 50 mg/mL ascorbic acid. Osteoblasts were treated with 100 nM PTH_1–34_ or vehicle for 2 hours. Etomoxir (MilliporeSigma) (10 μM) was added 60 minutes before Seahorse analysis. Metabolic flux analyses were performed with media containing 1 mM sodium pyruvate, 2 mM glutamine, 10 mM glucose, 200 nM insulin, and 200 μM oleic acid BSA. Analysis was performed using Agilent Seahorse XF Analyzer. Mito Stress test was performed using 1 μM oligomycin, 1 μM FCCP, and 1 μM rotenone/antimycin. The OCR was normalized to seeded cell number.

### Culture of primary osteoblasts.

Mouse osteoblasts were isolated from calvaria of 1- to 3-day-old neonates by serial digestion in 1.8 mg/mL collagenase (Worthington Biochemical). For in vitro deletion of Cpt2, osteoblasts isolated from Cpt2^fl/fl^ mice were infected with adenovirus encoding Cre recombinase or GFP (Vector Biolabs). A multiplicity of infection of 100 was used in all experiments, and gene deletion was confirmed by qPCR. Osteoblast differentiation was induced by supplementing α-MEM containing 10% serum with 10 mM β-glycerol phosphate and 50 μg/μL ascorbic acid. For matrix deposition studies, cells were treated with 100 μM PTH_1–34_ human acetate (BACHEM, H-4835) resuspended in culture-grade PBS (Corning) or PBS (vehicle) beginning on the day 7 differentiation, alternating osteogenic media change and treatment every day until the completion of the study on day 14. Cultures were stained with Picro Sirius Red Stain Kit (Abcam ab150681) or for alkaline phosphatase according to standard techniques.

### RNA sequencing.

Mouse osteoblasts were isolated and differentiated as described above. Cultures were serum-starved (0.1% FBS) for 6 hours on day 7 of differentiation. Osteoblasts were treated with 100 μM PTH_1–34_ for 24 hours. Total RNA was isolated with QIAGEN RNeasy Kit. Preparation of RNA library and transcriptome sequencing were conducted by Novogene Corporation Inc. The mRNA library was prepared using poly(A) enrichment. Sequencing was performed using NovaSeq 6000 PE150. RNA was of sufficient quality for all samples (RNA integrity number > 9). Raw reads were aligned to the reference *Mus musculus* (GRCM38) using Hisat2 (54). Binary read files (BAM) were generated and sorted using SAMtools. Transcript assembly was conducted using StringTie2 (55). Differential gene expression analysis was completed using DESeq2. Following that, a raw read matrix was generated using StringTie2 companion python script. Differential gene expression analysis was completed using DESeq2 using default read filtration settings. Genes with adjusted *P* value (FDR) < 0.05 and log_2_ fold-change deviating from 0 (log_2_FC > 0 or log_2_FC < 0) were considered differentially expressed. A GitHub page has been created for additional information on analysis (https://github.com/aa9gj/Riddle_2022_analysis; commit ID 2ca5030). The RNA-sequencing data are available in the NCBI Gene Expression Omnibus database (http://www.ncbi.nlm.nih.gov/geo) under the accession number GSE222173.

### In vitro oxidation studies.

Osteoblasts were cultured and differentiated in T25 cell culture flasks (Corning). Cultures were serum-starved (0.1% FBS) for 6 hours on day 7 of differentiation. Osteoblasts were treated with 100 μM PTH_1–34_ overnight. Oxidation by osteoblast cultures was measured in flasks with rubber stoppers equipped with center wells as previously described (25). Cultures were incubated at 37°C in media containing 0.5 mM l-carnitine, 0.2% BSA, and ^14^C-oleate (PerkinElmer) or ^14^C-d-glucose (PerkinElmer). Released CO_2_ was captured and counted by the addition of 1N perchloric acid to the reaction mixture and 1 M NaOH to the center well containing Whatman filter paper (Bio-Rad). The reaction was incubated overnight at 37°C, and the filter paper was placed in scintillation fluid and counted. Results were normalized to protein content.

### Gene expression and Western blotting.

Total RNA was extracted from cell cultures using TRIzol (Life Technologies). Reverse transcriptase reaction was completed using 1 μg of RNA and iScript cDNA Synthesis system (Bio-Rad). qPCR was carried out using iQ SYBR Green Supermix (Bio-Rad) using primer sequences obtained from PrimerBank (https://pga.mgh.harvard.edu/primerbank/). Cpt2 primers for qPCR specific for exon 4 were 5′ CCTGCTCGCTCAGGATAAACA 3′ (forward) and 5′ GTGTCTTCAGAAACCGCACTG 3′ (reverse). Pthr primers were 5′ CAGGCGCAATGTGACAAGC 3’ (forward) and 5′ TTTCCCGGTGCCTTCTCTTTC 3′ (reverse). Reactions were normalized to endogenous 18S reference transcripts.

For Western blotting, iWAT was collected and flash-frozen in liquid nitrogen 30 minutes after PTH or vehicle control administration. Western blot analyses were carried out according to standard technique using the primary antibodies phosphorylated HSL S660 (Cell Signaling Technology 4126S), HSL (Cell Signaling Technology 4107S), ATGL (Cell Signaling Technology 2138S), and ACTIN (Cell Signaling Technology 3700S).

### Statistics.

Quantitative data are shown as bar graphs produced using Prism (GraphPad Software). All results are expressed as mean ± SEM. Statistical analyses were performed using unpaired, 2-tailed Student’s *t* test or 1-wayANOVA tests followed by Tukey’s post hoc tests using Prism software. A *P* < 0.05 was considered significant.

### Study approval.

All mice were cared for under strict compliance with and with the approval of the Animal Care and Use Committee of the Johns Hopkins University School of Medicine and the University of Maryland School of Medicine.

## Author contributions

NSA, PK, MJW, and RCR were responsible for conceptualization; NSA, PK, AA, SO, ERR, MJW, and RCR were responsible for methodology; NSA, PK, SPK, ZL, AA, ND, SA, JK, JGGD, SO, ERR, and RCR were responsible for investigation; NSA, PK, and RCR were responsible for data curation; NSA, PK, MJW, and RCR were responsible for formal analysis; and NSA and RCR were responsible for writing.

## Version 1 02/02/2023

In-Press Preview

## Version 2 03/22/2023

Electronic publication

## Version 3 09/23/2025

In Figure 6D, the Ad∆Atgl panel was corrected (it was inadvertently duplicated from the control panel in the initial version)

## References

[1] Goltzman D (2018). Physiology of parathyroid hormone. Endocrinol Metab Clin North Am.

[2] Lee SK, Lorenzo JA (1999). Parathyroid hormone stimulates TRANCE and inhibits osteoprotegerin messenger ribonucleic acid expression in murine bone marrow cultures: correlation with osteoclast-like cell formation. Endocrinology.

[3] Rubin MR (2008). The natural history of primary hyperparathyroidism with or without parathyroid surgery after 15 years. J Clin Endocrinol Metab.

[4] Silverberg SJ (1999). A 10-year prospective study of primary hyperparathyroidism with or without parathyroid surgery. N Engl J Med.

[5] Shen V (1993). Loss of cancellous bone mass and connectivity in ovariectomized rats can be restored by combined treatment with parathyroid hormone and estradiol. J Clin Invest.

[6] Neer RM (2001). Effect of parathyroid hormone (1-34) on fractures and bone mineral density in postmenopausal women with osteoporosis. N Engl J Med.

[7] Dobnig H, Turner RT (1997). The effects of programmed administration of human parathyroid hormone fragment (1-34) on bone histomorphometry and serum chemistry in rats. Endocrinology.

[8] Jilka RL (1999). Increased bone formation by prevention of osteoblast apoptosis with parathyroid hormone. J Clin Invest.

[9] Wu X (2010). Inhibition of Sca-1-positive skeletal stem cell recruitment by alendronate blunts the anabolic effects of parathyroid hormone on bone remodeling. Cell Stem Cell.

[10] Dobnig H, Turner RT (1995). Evidence that intermittent treatment with parathyroid hormone increases bone formation in adult rats by activation of bone lining cells. Endocrinology.

[11] Finkelstein JS (1994). Parathyroid hormone for the prevention of bone loss induced by estrogen deficiency. N Engl J Med.

[12] Li X (2007). Determination of dual effects of parathyroid hormone on skeletal gene expression in vivo by microarray and network analysis. J Biol Chem.

[13] Sinha P (2016). Loss of Gsα in the postnatal skeleton leads to low bone mass and a blunted response to anabolic parathyroid hormone therapy. J Biol Chem.

[14] Abou-Samra AB (1992). Expression cloning of a common receptor for parathyroid hormone and parathyroid hormone-related peptide from rat osteoblast-like cells: a single receptor stimulates intracellular accumulation of both cAMP and inositol trisphosphates and increases intracellular free calcium. Proc Natl Acad Sci U S A.

[15] Singh AT (2005). G alpha12/G alpha13 subunits of heterotrimeric G proteins mediate parathyroid hormone activation of phospholipase D in UMR-106 osteoblastic cells. Endocrinology.

[16] Qiu T (2010). TGF-beta type II receptor phosphorylates PTH receptor to integrate bone remodelling signalling. Nat Cell Biol.

[17] Li C (2013). Disruption of LRP6 in osteoblasts blunts the bone anabolic activity of PTH. J Bone Miner Res.

[18] Bikle DD (2002). Insulin-like growth factor I is required for the anabolic actions of parathyroid hormone on mouse bone. J Bone Miner Res.

[19] Felix R (1978). Aerobic glycolysis in bone: lactic acid production by rat calvaria cells in culture. Am J Physiol.

[20] Neuman WF (1958). The mechanism of parathyroid function. J Lancet.

[21] Neuman WF (1978). Aerobic glycolysis in bone: lactate production and gradients in calvaria. Am J Physiol.

[22] Esen E (2015). PTH promotes bone anabolism by stimulating aerobic glycolysis via IGF signaling. J Bone Miner Res.

[23] Adamek G (1987). Fatty acid oxidation in bone tissue and bone cells in culture. Characterization and hormonal influences. Biochem J.

[24] Catherwood BD (1988). Growth of rat osteoblast-like cells in a lipid-enriched culture medium and regulation of function by parathyroid hormone and 1,25-dihydroxyvitamin D. J Bone Miner Res.

[25] Kim SP (2017). Fatty acid oxidation by the osteoblast is required for normal bone acquisition in a sex- and diet-dependent manner. JCI Insight.

[26] Weis BC (1994). Use of a selective inhibitor of liver carnitine palmitoyltransferase I (CPT I) allows quantification of its contribution to total CPT I activity in rat heart. Evidence that the dominant cardiac CPT I isoform is identical to the skeletal muscle enzyme. J Biol Chem.

[27] Sinha TK (1976). On the lipolytic action of parathyroid hormone in man. Metabolism.

[28] Wein MN, Kronenberg HM (2018). Regulation of bone remodeling by parathyroid hormone. Cold Spring Harb Perspect Med.

[29] Kobayashi T (2002). PTHrP and Indian hedgehog control differentiation of growth plate chondrocytes at multiple steps. Development.

[30] Sitnick MT (2013). Skeletal muscle triacylglycerol hydrolysis does not influence metabolic complications of obesity. Diabetes.

[31] Dube JJ (2015). Adipose triglyceride lipase deletion from adipocytes, but not skeletal myocytes, impairs acute exercise performance in mice. Am J Physiol Endocrinol Metab.

[32] Schoiswohl G (2015). Impact of reduced ATGL-mediated adipocyte lipolysis on obesity-associated insulin resistance and inflammation in male mice. Endocrinology.

[33] Wei J (2015). Glucose uptake and Runx2 synergize to orchestrate osteoblast differentiation and bone formation. Cell.

[34] Stegen S (2021). Glutamine metabolism in osteoprogenitors is required for bone mass accrual and PTH-induced bone anabolism in male mice. J Bone Miner Res.

[35] Justesen J (2001). Adipocyte tissue volume in bone marrow is increased with aging and in patients with osteoporosis. Biogerontology.

[36] Aaron N (2022). The implications of bone marrow adipose tissue on inflammaging. Front Endocrinol (Lausanne).

[37] Katzmarzyk PT (2012). Relationship between abdominal fat and bone mineral density in white and African American adults. Bone.

[38] Shin D (2014). Importance of fat mass and lean mass on bone health in men: the Fourth Korean National Health and Nutrition Examination survey (KNHANES IV). Osteoporos Int.

[39] Reid IR (1994). Volumetric bone density of the lumbar spine is related to fat mass but not lean mass in normal postmenopausal women. Osteoporos Int.

[40] Elefteriou F (2005). Leptin regulation of bone resorption by the sympathetic nervous system and CART. Nature.

[41] Kajimura D (2011). Genetic determination of the cellular basis of the sympathetic regulation of bone mass accrual. J Exp Med.

[42] Kajimura D (2013). Adiponectin regulates bone mass via opposite central and peripheral mechanisms through FoxO1. Cell Metab.

[43] Zou W (2019). Congenital lipodystrophy induces severe osteosclerosis. PLoS Genet.

[44] Li Z (2022). Lipolysis of bone marrow adipocytes is required to fuel bone and the marrow niche during energy deficits. Elife.

[45] Maridas DE (2019). Progenitor recruitment and adipogenic lipolysis contribute to the anabolic actions of parathyroid hormone on the skeleton. FASEB J.

[46] Balani DH (2017). Parathyroid hormone regulates fates of murine osteoblast precursors in vivo. J Clin Invest.

[47] Fan Y (2017). Parathyroid hormone directs bone marrow mesenchymal cell fate. Cell Metab.

[48] Lee J (2016). Loss of adipose fatty acid oxidation does not potentiate obesity at thermoneutrality. Cell Rep.

[49] Lee J (2015). Adipose fatty acid oxidation is required for thermogenesis and potentiates oxidative stress-induced inflammation. Cell Rep.

[50] Zhang M (2002). Osteoblast-specific knockout of the insulin-like growth factor (IGF) receptor gene reveals an essential role of IGF signaling in bone matrix mineralization. J Biol Chem.

[51] Eguchi J (2011). Transcriptional control of adipose lipid handling by IRF4. Cell Metab.

[52] Bouxsein ML (2010). Guidelines for assessment of bone microstructure in rodents using micro-computed tomography. J Bone Miner Res.

[53] Dempster DW (2013). Standardized nomenclature, symbols, and units for bone histomorphometry: a 2012 update of the report of the ASBMR Histomorphometry Nomenclature Committee. J Bone Miner Res.

[54] Kim D (2015). HISAT: a fast spliced aligner with low memory requirements. Nat Methods.

[55] Pertea M (2015). StringTie enables improved reconstruction of a transcriptome from RNA-seq reads. Nat Biotechnol.

