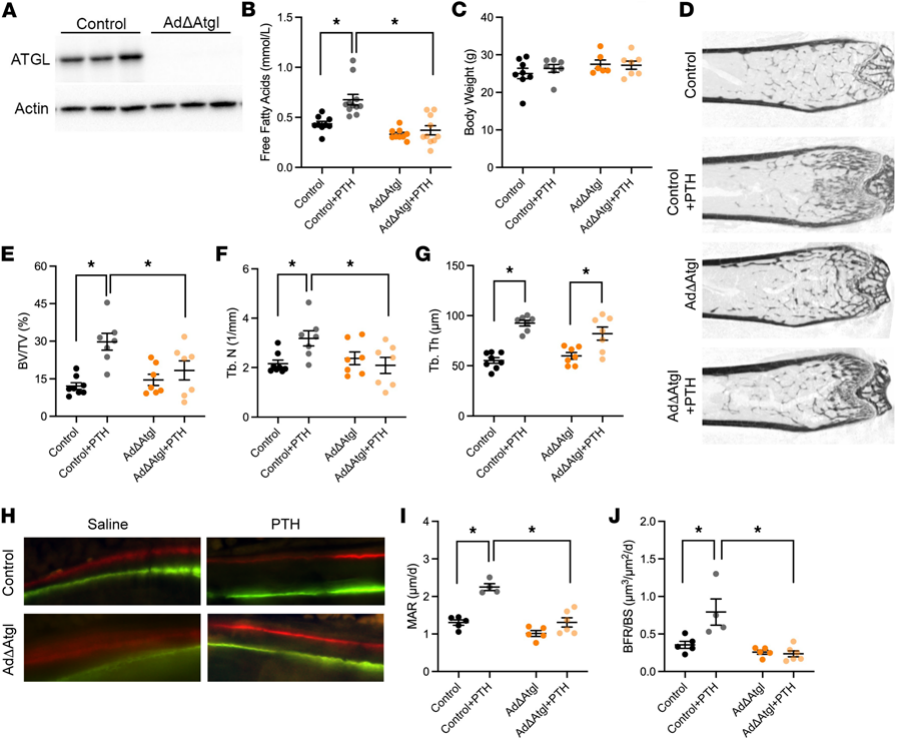# Mitochondrial β-oxidation of adipose-derived fatty acids by osteoblasts fuels parathyroid hormone–induced bone formation

**DOI:** 10.1172/jci.insight.198337

**Published:** 2025-09-23

**Authors:** Nathalie S. Alekos, Priyanka Kushwaha, Soohyun P. Kim, Zhu Li, Abdullah Abood, Naomi Dirckx, Susan Aja, Joe Kodama, Jean G. Garcia-Diaz, Satoru Otsuru, Elizabeth Rendina-Ruedy, Michael J. Wolfgang, Ryan C. Riddle

Original citation: *JCI Insight*. 2023;8(6):e165604. https://doi.org/10.1172/jci.insight.165604

Citation for this corrigendum: *JCI Insight*. 2025;10(18):e198337. https://doi.org/10.1172/jci.insight.198337

The authors recently became aware that in Figure 6D, the AdΔAtgl panel was inadvertently duplicated from the control panel during the revision process. The correct panel is provided below. The HTML and PDF files have been updated.

The authors regret the error.

## Figures and Tables

**Figure 6:**